# Simultaneous occurrence of Pyoderma Gangrenosum and Palmoplantar Pustular Psoriasis: Is it an association or coincidental findings?

**DOI:** 10.1002/ccr3.3544

**Published:** 2020-11-18

**Authors:** Chapagain Pukar, Agrawal Sudha, Paudyal Punam

**Affiliations:** ^1^ Department of Dermatology and Venereology BP Koirala Institute of Health Sciences Dharan Nepal; ^2^ Department of Pathology BP Koirala Institute of Health Sciences Dharan Nepal

**Keywords:** dermatology, neutrophilic dermatosis, Plantar Pustular Psoriasis, Pyoderma gangrenosum

## Abstract

Pyoderma Gangrenosum and Palmoplantar Pustular Psoriasis share the common pathogenesis, histological features, age group, and female preponderance that suggests a common etiological link.

## Graphical abstract

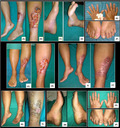

## 1 INTRODUCTION

Simultaneous occurrence of Pyoderma Gangrenosum and Palmoplantar Pustular Psoriasis: Is it an association or coincidental findings? A female presented with Palmoplantar Pustular psoriasis (PPP) followed by Pyoderma gangrenosum (PG) on bilateral legs. Complete remission was achieved with methotrexate and prednisolone. Both dermatoses have female preponderance, similar immuno‐pathologic mechanisms and rapid treatment response, suggests a possible etiological relation between the two and not a coincidental finding.

Pyoderma gangrenosum is a rare noninfectious neutrophilic dermatosis of unknown etiology, characterized clinically by chronic and recurrent cutaneous ulcers with a necrolytic border and is commonly associated with underlying systemic disease. About 33%‐50% of the patients are known to have various underlying diseases such as inflammatory bowel disease (IBD), hematological malignancies, and rheumatoid arthritis.^1^, ^2^ Whereas palmoplantar pustulosis (PPP) presents with erythematous and scaly plaques studded with sterile pustules localized on the palms and/or soles and has a chronic and relapsing course. It is commonly associated with psoriasis vulgaris and or psoriatic arthritis.^3^

Although the pathogenesis of PG and PPP is poorly understood, recent studies have suggested that dysregulation of the innate immunity involving several common cytokines such as tumor necrosis factor‐a (TNF‐α), interleukin (IL)‐17, and (IL)‐23 may play important role in the development of both PG and PPP and share a common characteristic histological feature of neutrophilic infiltration in skin.^4^, ^5^, ^6^ Psoriasis and psoriatic arthritis have been found to be associated in up to 10% of PG patients^7^ but the coexistence of PG and PPP in the same patient has been reported only in 5 cases in the literature till date^8^, ^9^, ^10^ and all were reported from Japan. We herein report a female patient with PPP who developed PG on her lower leg as a first case report other than in Japanese population and tried to justify the coexistence of both the conditions based on the review of the literature.

## 2 CASE REPORT

A 45‐year‐old Nepalese female presented with 4 months history of moderately itchy erythematous plaque on bilateral soles with few discrete pustules and yellow‐brown crust. Three months after onset of lesions, she developed multiple pustules on erythematous base over the extensor aspect of left leg associated with moderate pain. These pustules ruptured to form ulcers over 3‐4 days which coalesced rapidly over 1‐2 weeks to form a large ulcer of irregular shape measuring 10 × 8 cm^2^ with undermined edge and nonfoul smelling scanty serous discharge on floor with hyperpigmented surrounding skin. There was no history of trauma prior to the onset of lesion, significant weight loss, fever, arthralgia, or gastrointestinal symptoms and was a nonsmoker.

**FIGURE 1:**
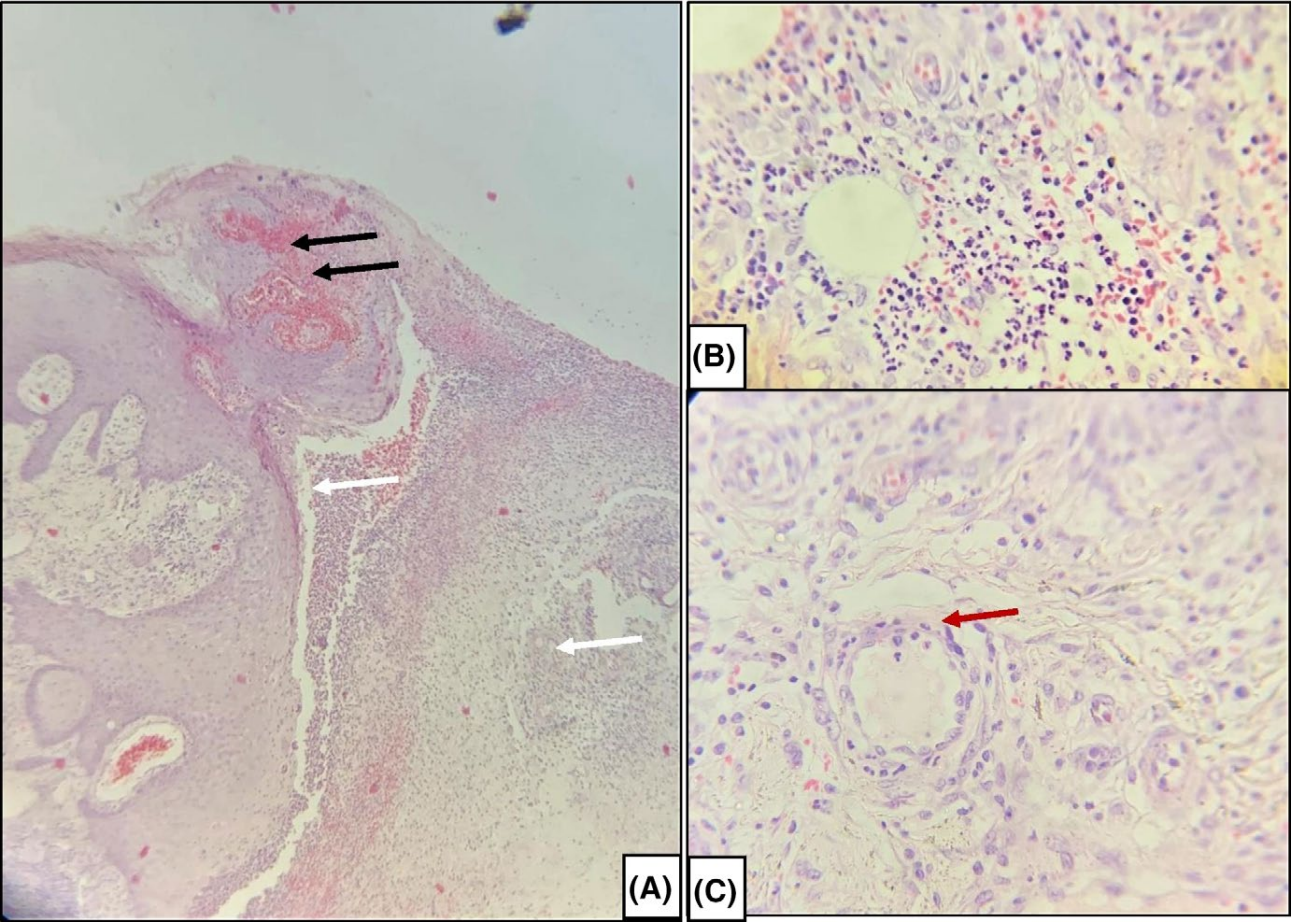
A, (10×) H &amp; E stain showing hemorrhagic ulcer (black arrows) adjoining an intact epidermis with large necrotizing suppurative dermal inflammation (white arrows) (B) (40×) H &amp; E stain showing dense neutrophilic infiltrate in the dermis with extravasation of Red blood cells (C) (40×) H &amp; E stain (red arrows) showing endothelial swelling with neutrophilic infiltration of blood vessel wall

The biopsy specimen from the edge of the leg ulcer showed a hemorrhagic ulcer adjoining an intact epidermis with large necrotizing suppurative dermal inflammation, leukocytoclastic vasculitis within central suppurative zone, angiocentric and intraneural lymphocyte infiltration and uninvolved zones showed normal hair follicles and adnexal structures surrounded by lymphocytes (Figure 1A‐C). Special stains were negative for organism, tuberculous bacilli, mucin, or fibrin.

Other laboratory tests, including complete blood count, liver and renal functions, erythrocyte sedimentation rate and chest X‐ray were unremarkable. A culture from the ulcer did not demonstrates any pathogenic organisms. A diagnosis of classical ulcerative type of pyoderma gangrenosum with palmoplantar pustulosis was made. She was treated with a tapering dose of oral prednisolone (50 mg/day) and the ulcer healed within 1 and 1/2 months to leave an atrophic scar. Then the patient was lost to follow up.

**FIGURE 2:**
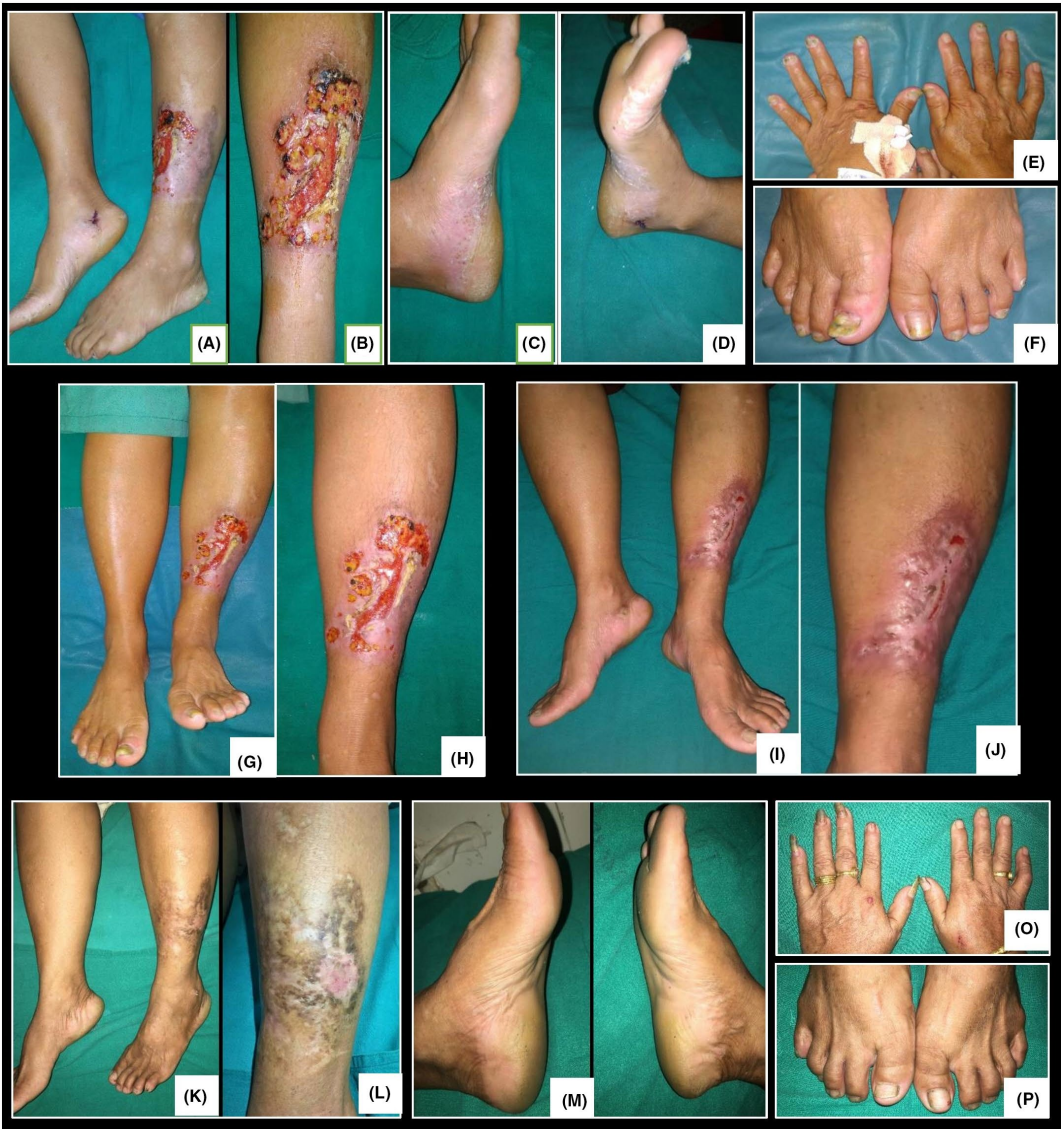
A and B, showing irregular undermined ulcer with atrophic plaque on dorsum of left foot, (C and D) erythematous plaque with yellow‐brown crust, (E and F) onycholysis and subungual hyperkeratosis, (G and H) 1 wk after starting treatment, (I and J) 4 wk after starting treatment and (M‐P) 9 mo after stopping the treatment

Four months later, she again developed multiple pustules over previously healed atrophic scar with similar progression as in the previous episode to form a larger ulcer. On examination, an irregular shaped ulcer approximately 7 × 4 cm^2^ was present over lower 1/3rd of dorsum of left leg with undermined edge and hyperpigmented border with scanty serous discharge, granulation tissue, and adherent yellowish crust on the floor. Similar smaller ulcers approximately 1‐2 cm^2^ were present on the periphery of larger ulcer. The ulcer was tender with mild induration of the borders. An atrophic plaque with hyperpigmented margin was present on the dorso‐lateral aspect of leg surrounding the ulcer (Figure 2A,B). Similarly, on bilateral soles, well defined erythematous plaque with semi adherent whitish scales with multiple adherent yellow‐brown crust and few pustules was present (Figure 2C,D). Nail examination revealed yellowish discoloration, distal onycholysis, few pits on almost all nail plates of bilateral hands and feet with subungual hyperkeratosis of great toes (Figure 2E,F).

**FIGURE 3:**
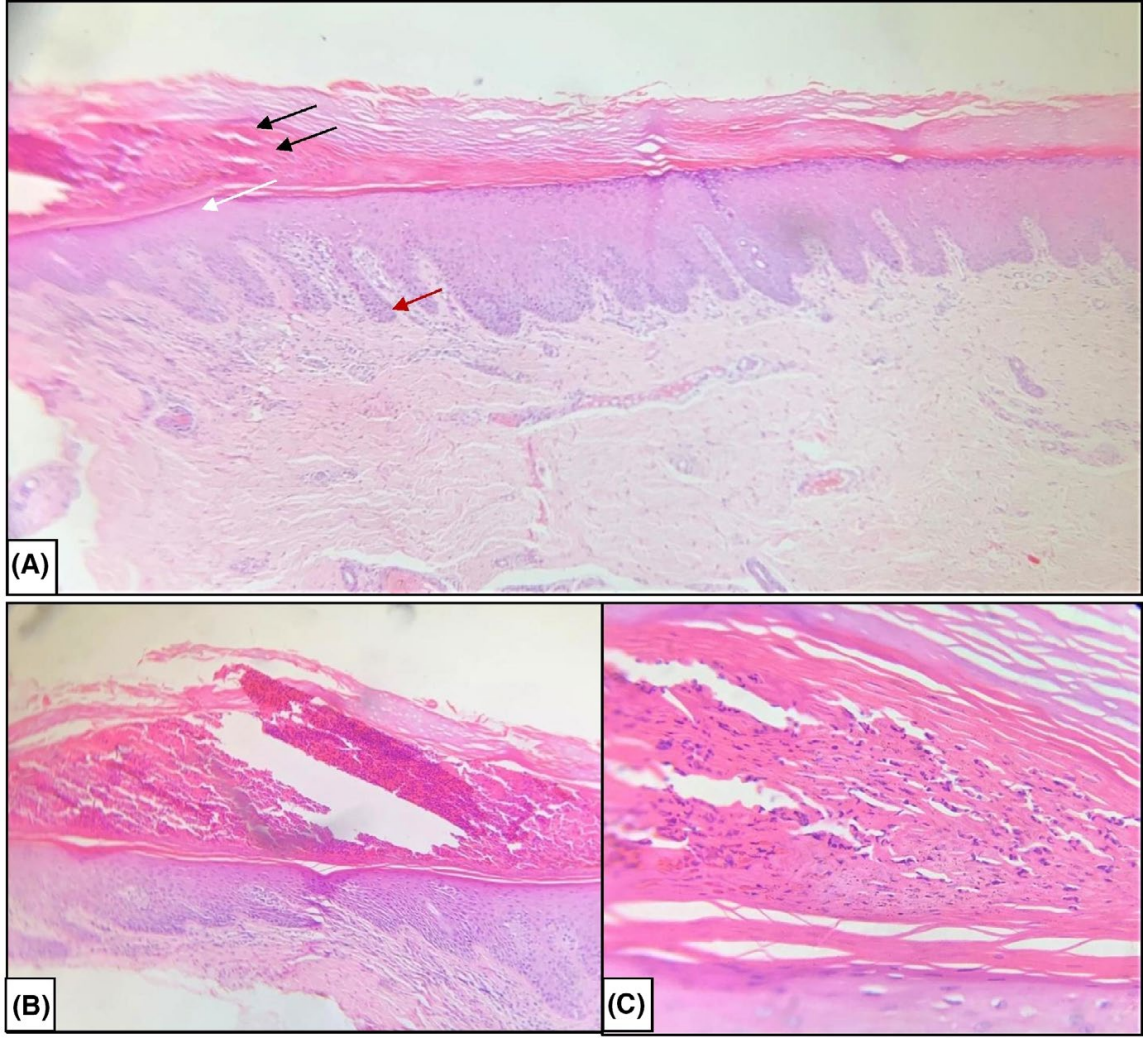
A, (10×) H &amp; E stain showing hyperkeratosis with parakeratosis with munro's microabscess (black arrows) with hypogranular layer (white arrow) with psoriasiform hyperplasia of epidermis (red arrow) (B) (10×) H &amp; E stain showing munro microabscess with underlying hypogranular layer (C) 100× magnification showing neutrophil and neutrophilic dusts in stratum corneum (munro microabsess)

Biopsy of the erythematous plaque on right sole showed hyperkeratosis with parakeratosis with munro's microabscess and psoriasiform hyperplasia of epidermis (Figure 3A‐C).

Laboratory tests including complete blood count, liver and renal functions, erythrocyte sedimentation rate and chest X‐ray were unremarkable. The culture of wound swab was sterile. The patient was admitted in the dermatology ward and was given 8 mg dexamethasone intravenously for 5 days, which was later changed to oral prednisolone 80 mg per day along with wound care. The patient was also started on tablet methotrexate 15 mg per week. The ulcer showed rapid improvement over 1 week with 30%‐40% improvement in pain and discharge and 15% improvement in the size of the ulcer (Figure 2G,H). The ulcer of PG and lesions of PPP showed about 90% improvement after 4 weeks of treatment (Figure 2I,J). The dose of prednisolone was tapered weekly 10 mg for 4 weeks and then every 2 weeks till 10 mg. The dose of methotrexate was increased to 20 mg per week after 6 weeks and then tapered to 17.5 mg/week dose which she continued for 4 weeks. The patient was unable to come for follow up and she stopped all medication after 20 weeks of initiating treatment. However, she visited OPD after 9 months and was on complete remission with healed scar on the site of previous ulcer (Figure 2K‐N) with significant improvement on nails (Figure 2O,P).

## 3 DISCUSSION

Pyoderma gangrenosum is a rare inflammatory skin disorder occurring most commonly between 20‐50 years of age^11^ with females comprising of 59‐76% of cases.^12^ PG is clinically classified into 4 types, *that is,* ulcerative, bullous, pustular, and vegetative types. Ulcerative PG is the most common type that is characterized by appearance of small follicular pustules appear initially that rapidly forms abscess and ulcerates. These ulcers enlarge rapidly to form irregular ulcers undermined edge, raised edematous and violaceous borders with necrotic tissues and scanty discharge on floor. The most frequently involved site of PG is the lower legs; however, any other sites can also be involved. ^11^

PG is associated with other diseases in 33‐50% of cases.^1^, ^2^ The most frequent disease associations include Inflammatory Bowel Disease (IBD) present in 20‐30% of cases. Rheumatoid arthritis and other seronegative arthritis's occur in about 10% of cases, hematological malignancy or monoclonal gammopathy in approximately 5% and other visceral malignancies in 5%.^7^, ^11^ The association of pyoderma gangrenosum with psoriasis and psoriatic arthritis has been found to be approximately 10%.^7^

Palmoplantar pustulosis is a chronic inflammatory dermatosis characterized by erythematous and scaly plaques studded with sterile pustules on the palms and/or soles. It occurs most commonly between the ages of 30 and 50 years ^12^with a female predominance ratio of 5:1.^13^ The associated nail changes are seen in 30%‐76% of cases of PPP. The most common finding is onycholysis, followed by pitting and destruction of the nail. The other nail changes include scale, subungual hyperkeratosis, subungual pustulation, indention, transverse and longitudinal ridging, curvature abnormalities, discoloration, splinter hemorrhage, and thickening of the nail.^14^ In our patient yellowish discoloration, distal onycholysis and a few pits were found in all most all nail plates of bilateral hands and feet and subungual hyperkeratosis in the great toes.

Palmoplantar pustulosis is most commonly associated with psoriasis and or psoriatic arthritis in 24%‐84.21% of cases followed by autoimmune thyroid disease in 20%‐40% of cases and it is the commonest cutaneous manifestation of SAPHO syndrome (synovitis, acne, pustulosis, hyperostosis, and osteitis).^3^, ^15^ Approximately 10% of patients with palmoplantar pustulosis suffer from osteoarticular symptoms, termed pustulotic arthro‐osteitis (PAO), which most commonly affects the stern costoclavicular joints, followed by the spine and sacroiliac joints.^10^

The pathogenesis of both palmoplantar pustulosis remains unclear but they are considered as a spectrum of common disorder involving several common cytokines such as tumor necrosis factor‐a (TNF‐α), interleukin (IL)‐17, and (IL)‐23 and share a common histological feature of neutrophilic infiltration.^3^, ^4^, ^5^ Recent studies have demonstrated that TNF‐a, IL‐17, and IL‐23 are increased in lesional skin of PG.^4^, ^5^, ^6^ Upregulation of TNF‐a, IL‐17, and IL‐23 also have been observed in PPP skin samples. In 2014, Ohtsuka and Yamamoto observed IL‐17‐positive lymphocytes in both of the biopsy samples obtained from a leg ulcer and plantar pustule.^10^ These findings suggest that the T‐helper (Th) 17‐associated cytokines may play crucial roles in both PG and PPP. Many patients with PG as well as PPP responded well to biologic agents such as anti‐TNF‐a and anti‐IL‐12/23p40 antibodies.^16^, ^17^

Although association between plaque psoriasis and psoriatic arthritis is observed, coexistence of PG and Palmoplantar pustular psoriasis (PPP) is rarely reported in literature. Occurrence of multiple autoinflammatory diseases simultaneously in one patient has been the subject of several reports. This phenomenon may result from similar etiologic pathways involved in the development of the autoinflammatory conditions. Similarly, coexistence of PG and PPP may be implicated by similar etiopathogenesis of both conditions. Only five cases have been reported till date in Japanese population and our is the 6th case in the literature and first case report from Nepal. The genetic predisposition may influence the susceptibility of PG and PPP; however, the genetic background of PPP is complex. There may be some ethnic association and may be similarities between Japanese and ours case, but we were not able to do the genetic study. Certain stimuli are required to provoke PG. In all 5 reported cases, PPP was preceded 1 month to 30 years to give rise to the onset of PG lesion.10 In our case also, the lesions of PPP developed 4 months prior to the development of PG lesion. PPP may be a triggering factor for PG and active lesions of PPP is needed for the occurrence of PG. Like in our case, PPP and PG recurred after the stopping of the oral prednisolone.

The first line of treatment of PG is systemic corticosteroids. However, for the refractory cases, other immunosuppressive and immunomodulatory drugs, such as cyclosporine, azathioprine, methotrexate, thalidomide, tacrolimus, mycophenolate mofetil, and recently biologics are also used. Methotrexate has been found to reduce the neutrophil chemotaxis, suggesting a role for its use in pyoderma gangrenosum. However, no therapeutic standards have been established for controlling PPP. Various treatments including methotrexate have been used to treat. The patient's condition may be well controlled with low‐dose regimen of methotrexate. It may be effective in the treatment of nail complications, reduce the serious adverse effects of corticosteroid, cheaper than retinoid and azathioprine. Therefore, we gave in our patient a short duration of oral prednisolone and maintained with the low dose of methotrexate with good response to prevent the recurrence of the conditions and hoping to get a good response in nail changes of PPP.

In conclusion, PG and PPP share the common pathogenesis with histological features, common age group and female preponderance suggest a common etiological link. Further research is required to ensure the etiological relationship between PG and PPP.

## CONFLICT OF INTEREST

None declared.

## AUTHOR'S CONTRIBUTION

CP: preparation and literature review. AS: Idea, concept and editing. PP: Histopathology reporting.

## ETHICAL APPROVAL

This case report was ethically approved by Institutional Review Committee (IRC) of BP Koirala Institute of Health Sciences (BPKIHS).

## CONSENT STATEMENT

Patient provided written consent for publication of this case report. It is available upon request.

## Data Availability

Data will be made available upon request.
